# Differential Parametric Formalism for the Evolution of Gaussian States: Nonunitary Evolution and Invariant States

**DOI:** 10.3390/e22050586

**Published:** 2020-05-23

**Authors:** Julio A. López-Saldívar, Margarita A. Man’ko, Vladimir I. Man’ko

**Affiliations:** 1 Instituto de Ciencias Nucleares, Universidad Nacional Autónoma de México, Apdo. Postal 70-543, Ciudad de México 04510, Mexico; 2 Moscow Institute of Physics and Technology (State University), Institutskii per. 9, Dolgoprudnyi, 141700 Moscow Region, Russia; mankovi@lebedev.ru; 3 Lebedev Physical Institute, Leninskii Prospect 53, 119991 Moscow, Russia; mankoma@lebedev.ru; 4 Department of Physics, Tomsk State University, Lenin Avenue 36, 634050 Tomsk, Russia

**Keywords:** Gaussian states, integrals of motion, parametric processes, nonunitary evolution, quantization, invariant states, covariance matrix

## Abstract

In the differential approach elaborated, we study the evolution of the parameters of Gaussian, mixed, continuous variable density matrices, whose dynamics are given by Hermitian Hamiltonians expressed as quadratic forms of the position and momentum operators or quadrature components. Specifically, we obtain in generic form the differential equations for the covariance matrix, the mean values, and the density matrix parameters of a multipartite Gaussian state, unitarily evolving according to a Hamiltonian $\hat{H}$. We also present the corresponding differential equations, which describe the nonunitary evolution of the subsystems. The resulting nonlinear equations are used to solve the dynamics of the system instead of the Schrödinger equation. The formalism elaborated allows us to define new specific invariant and quasi-invariant states, as well as states with invariant covariance matrices, i.e., states were only the mean values evolve according to the classical Hamilton equations. By using density matrices in the position and in the tomographic-probability representations, we study examples of these properties. As examples, we present novel invariant states for the two-mode frequency converter and quasi-invariant states for the bipartite parametric amplifier.

## 1. Introduction

The study of Gaussian states has been of essential interest in the last few decades. These types of states, associated with classical random fields, were considered as a possibility to connect covariance matrices of the states as quantum density matrices and, with this definition, to study the quantum–classical relation of randomness with the quantization procedure [1,2]. The problems of the new developments of the foundations of quantum mechanics and applications of new results in quantum information and quantum probabilities, as well as in areas like mathematical finance and economics have attracted the attention of the researchers; they are intensely discussed in the literature [3,4,5]. An important role in this development is played by discussing the problems that appeared from the very beginning of quantum mechanics, like the notion of quantum system states and the interpretation of the states associated in the conventional formulation of quantum mechanics with Hilbert space vectors and density operators, using the quasiprobability distributions and the probability distributions containing the complete information on quantum states. There exists increasing interest in quantum foundations since a deeper understanding of the essence and formalism of quantum theory is needed for the development of quantum technologies and the possibilities to extend the applications of quantum formalism in physics to all other areas of science like the economy, finance, and social disciplines.

Some examples of the Gaussian states of quantum fields as the coherent, squeezed, and thermal light states are regularly used in the theoretical and experimental framework of quantum mechanics, optics, information, and computing. The use of these states in quantum information has been of particular importance [6,7,8]. One can list some of the most recent applications of the use of Gaussian systems: it has been demonstrated [9] that it is not possible to distill more entanglement from a bipartite Gaussian state, using local Gaussian transformations. In [10], several properties of the purity of Gaussian states were found. The connection between the symplectic invariants of bipartite Gaussian states, the von Neumann entropy, and the mutual information was established in [11]. The extremality of entanglement measures and secret key rates for Gaussian states was observed in [12]. It was shown [13] that Gaussian attacks are characterized by an optimum efficiency against eavesdropping protocols. Quantum illumination of a target using Gaussian light states was explored by Tan et al. [14]. A quantum discord for systems of continuous variables, such as Gaussian states, was implemented in [15]. In [16], an invariant describing the nonclassicality in a two-mode Gaussian state was reported. The entanglement of *m* modes with other *n* modes of a Gaussian multipartite system was treated in [17]. The linear response for systems close to steady-states under Gaussian processes was obtained in [18]. The optimal measurement of the fidelity of multimode Gaussian states was studied in [19]. On the other hand, the study of Gaussian wave packets by nonlinear differential equations, as the Riccati equation, was done in [20,21,22]. Several coherent states have been defined by the use of quadratic operators [23]. The behavior of different quantities as covariances in thermal relaxation phenomena was also studied in [24].

The aim of this work is to present a new way to characterize the dynamics of Gaussian states using the differential equations for the parameters, which determine their continuous variable density matrices. The proposed method makes use of the integrals of the motion of such systems, and it can be used to clarify new aspects of multimode Gaussian quantum states, such as an explicit form of the nonunitary evolution of the states of subsystems and the existence of invariant states with constant covariance matrices and mean values.

The time evolution of a quantum system was first established by Schrödinger [25]. The dynamics of the system given by a Hamiltonian operator $\hat{H}$ for a pure state |ψ(t)〉 must follow the Schrödinger equation:

$$\hat{H}|\psi \left(t\right)\rangle =i\hbar \frac{\partial }{\partial t}|\psi \left(t\right)\rangle \;;$$

this expression corresponds to a second-order differential equation in the position representation. In the case of an arbitrary state represented by the density matrix $\hat{\rho }\left(t\right)$, which cannot be pure [26,27], the evolution is determined by the von Neumann equation:

$$i\hbar \frac{\partial }{\partial t}\hat{\rho }\left(t\right)=\left[\hat{H},\hat{\rho }\left(t\right)\right]\;;$$

the general solution of this equation is given by the unitary transform $\hat{U}\left(t\right)$, i.e., $|\psi \left(t\right)\rangle =\hat{U}\left(t\right)|\psi \left(0\right)\rangle$ or $\hat{\rho }\left(t\right)=\hat{U}\left(t\right)\hat{\rho }\left(0\right){\hat{U}}^{†}\left(t\right)$, where |ψ(0)〉 and $\hat{\rho }\left(0\right)$ describe the system at time t=0. Furthermore, it is a common knowledge that, when the system interacts with an environment, its dynamics is described by the master equation [28,29,30].

The Gaussian states can be determined by their covariance matrix σ and mean values $\langle q_{j}\rangle$ and $\langle p_{j}\rangle$. This property also implies that the evolution of a Gaussian state can be obtained, if the time dependence of these parameters is known. In this work, we review the differential equations that the covariance matrix and the mean values satisfy [31,32]; employing these results, we can define differential equations for the density matrix parameters of a general multimode state satisfying these equations and then use the equations to discuss some physical characteristics of the unitary and nonunitary evolutions of Gaussian states.

This paper is organized as follows.

In Section 2, the evolution of non-pure Gaussian states for a one-dimensional quadratic Hamiltonian is presented. To obtain this evolution, we make use of the derivatives of the covariance matrix, the mean values, and the parameters of the density operator; also, we define and obtain invariant states for this system. The generalization of these results to the case of a multidimensional quadratic system is explored in Section 3. Examples of the application of the general results to the nonunitary evolution of the subsystems of a two-mode state, as well as the definition of invariant and quasi-invariant states are given in Section 4. Furthermore, in Section 5, we obtain new invariant states for the frequency converter and quasi-invariant states for the parametric amplifier. The detection of these invariant states using the quantum tomographic representation of the states is discussed for single-mode Gaussian states in Section 5 and for the bipartite system in Section 6; in these sections, the correspondence between the time-independent states and thermal density matrices is mentioned. Finally, we give our conclusions.

## 2. One-Dimensional Quantum Quadratic Hamiltonian and Its Linear Invariant Operators

In this section, we analyze some properties of the one-dimensional quadratic Hamiltonian. In particular, we are interested in the invariant operators, which in the quadratic case happen to be linear in the quadrature operators $\hat{p}$ and $\hat{q}$.

The most general (in a unit system where ℏ=m=1), one-dimensional quantum quadratic Hamiltonian can be obtained in terms of the quadrature operators $\hat{p}$ and $\hat{q}$ as follows:

(1) $$\hat{H}=\left(\hat{p},\hat{q}\right)\left(\begin{array}{cc} \omega_{1}\left(t\right) & \omega_{2}\left(t\right)\\ \omega_{2}\left(t\right) & \omega_{3}\left(t\right) \end{array}\right)\left(\begin{array}{c} \hat{p}\\ \hat{q} \end{array}\right)+\left(\hat{p},\hat{q}\right)\left(\begin{array}{c} \delta_{1}\\ \delta_{2} \end{array}\right)\;,$$

where the parameters $\omega_{1}\left(t\right)$, $\omega_{2}\left(t\right)$, $\omega_{3}\left(t\right)$, and $\delta_{1,2}$ are real functions of time. The dynamics associated with this Hamiltonian can be solved by different methods. One of them is the method of time-dependent invariants (integrals of motion) [33,34]. These invariants are quantum operators $\hat{R}\left(t\right)$, whose total time derivative is equal to zero $\frac{d\hat{R}\left(t\right)}{dt}=0$. In the quadratic case, it is known that there exist invariants linearly depending on the quadrature operators $\hat{p}$ and $\hat{q}$, i.e., $\hat{R}\left(t\right)=\lambda_{1}\left(t\right)\hat{p}+\lambda_{2}\left(t\right)\hat{q}+\lambda_{3}\left(t\right)$.

By substituting this expression into the von Neumann equation, which determines the dynamics of $\hat{R}$, i.e., $\frac{d\hat{R}\left(t\right)}{dt}=\frac{i}{\hbar }\left[\hat{H}\left(t\right),\hat{R}\left(t\right)\right]+\frac{\partial \hat{R}\left(t\right)}{\partial t}=0$, one can show that $\hat{R}\left(t\right)$ is an invariant operator, if the following differential equations are satisfied:

(2) $$\begin{array}{c} {\dot{\lambda }}_{1}=2\left(\omega_{2}\lambda_{1}-\omega_{1}\lambda_{2}\right)\;,\;{\dot{\lambda }}_{2}=2\left(-\omega_{2}\lambda_{2}+\omega_{3}\lambda_{1}\right)\;,\;{\dot{\lambda }}_{3}=\delta_{2}\lambda_{1}-\delta_{1}\lambda_{2}\;. \end{array}$$

We point out that parameters $\lambda_{1,2}$ can be obtained by solving the classical Hamilton equations:

(3) $$\begin{array}{c} \dot{p}=-2\left(\omega_{2}p+\omega_{3}q\right)-\delta_{2}\;,\;\dot{q}=2\left(\omega_{2}q+\omega_{1}p\right)+\delta_{1}\;. \end{array}$$

with $\delta_{1}=\delta_{2}=0$. To show this, one can see that the differential equations for $\lambda_{1,2}$ correspond to the classical equations with the substitution $\lambda_{1}\to q$ and $\lambda_{2}\to -p$; in other words, they correspond to the time inversion of the classical equations. In the case of the differential equation for $\lambda_{3}$, one can show, in view of the Hamilton equations, that it corresponds to the classical Lagrangian (with $\delta_{1,2}\neq 0$) plus the time variation of the function pq of the system, that is,

(4) $${\dot{\lambda }}_{3}=-2L+\dot{L}\;,$$

where $L=p\dot{q}-H$ and L=pq. From these identifications, one can conclude that the classical dynamics given by the Hamilton equation or the equation of motion can lead to the solution of the quantum dynamics given by the Hamiltonian operator of Equation (2). For example, one can derive the propagator of the system $G\left(x,x^{\prime },t\right)=\langle x|\hat{U}\left(t\right)|x^{\prime }\rangle$ using each one of the solutions of the classical problem [35].

### 2.1. Dynamics of Non-Pure States

Here, we demonstrate that the dynamics of a generic Gaussian state, which may not be pure, can be given by solving differential equations for the covariance matrix or the density matrix parameters. We show that these differential equations imply the invariance of the determinant of the covariance matrix, when the time evolution is unitary.

As discussed above, the propagator of the system can be obtained using the time-dependent invariants resulting from the solution to Equation (2) for two sets of initial conditions: $\lambda_{1}\left(0\right)=1$, $\lambda_{2}\left(0\right)=0$, $\lambda_{3}\left(0\right)=0$ and $\lambda_{1}\left(0\right)=0$, $\lambda_{2}\left(0\right)=1$, $\lambda_{3}\left(0\right)=0$. These two sets define two different invariants called $\hat{P}$ and $\hat{Q}$, respectively, which can be written as:

(5) $$\left(\begin{array}{c} \hat{P}\\ \hat{Q} \end{array}\right)=\Lambda \left(\begin{array}{c} \hat{p}\\ \hat{q} \end{array}\right)+\left(\begin{array}{c} \lambda_{3}\\ \lambda_{6} \end{array}\right)\;,\;\mathrm{with}\;\Lambda =\left(\begin{array}{cc} \lambda_{1} & \lambda_{2}\\ \lambda_{4} & \lambda_{5} \end{array}\right)\;,$$

where $\lambda_{4,5,6}$ satisfy the same differential equations as $\lambda_{1,2,3}$, respectively, with the different sets of initial conditions mentioned above. The operators $\hat{P}$ and $\hat{Q}$ fulfill the commutation relation $[\hat{Q},\hat{P}]=i$, implying the relation $\lambda_{1}\lambda_{5}-\lambda_{2}\lambda_{4}=1$ and the fact that the matrix Λ is symplectic.

Furthermore, they can be related to operators $\hat{p}$ and $\hat{q}$ through the evolution operator as follows:

$$\hat{P}=\hat{U}\hat{p}\;{\hat{U}}^{†}\;,\;\hat{Q}=\hat{U}\hat{q}\;{\hat{U}}^{†}\;;$$

it is not difficult to show [33,34] that these expressions can be used to obtain the propagator of the system $G\left(x,x^{\prime },t\right)=\langle x|\hat{U}\left(t\right)|x^{\prime }\rangle$, which reads:

(6) $$\begin{array}{lll} G(x,x^{\prime },t) & = & \frac{1}{\sqrt{-2\pi i\lambda_{4}}}\;\exp\left\{-\frac{i}{2\lambda_{4}}[\lambda_{5}x^{2}-2xx^{\prime }+\lambda_{1}{x^{\prime }}^{2}+2x\lambda_{6}+2x^{\prime }\left(\lambda_{3}\lambda_{4}-\lambda_{1}\lambda_{6}\right)\right.\\ & & \left.\;\;\;\;+\lambda_{1}\lambda_{6}^{2}-2\lambda_{4}\int_{0}^{t}{\dot{\lambda }}_{3}\lambda_{6}d\tau ]\right\}, \end{array}$$

which we immediately identify as a Gaussian function.

In view of this propagator, the dynamics of any initial state in the position representation can be found by the integration of the propagator and the wave function of the initial state. In this work, we will suppose the case of the initial state given by a generic mixed Gaussian system with the density matrix in the position representation $\rho \left(x,x^{\prime },t=0\right)=\langle x|\hat{\rho }|x^{\prime }\rangle$ equal to:

(7) $$\rho \left(x,x^{\prime },t=0\right)=N\exp\left\{-a_{1}x^{2}+a_{12}xx^{\prime }-a_{1}^{*}{x^{\prime }}^{2}+b_{1}x+b_{1}^{*}x^{\prime }\right\}\;,$$

with complex parameters $a_{1}=a_{1R}+ia_{1I}$ and $b_{1}=b_{1R}+ib_{1I}$ and the real parameter $a_{12}\in R$, which also satisfy the integrability conditions $a_{1R}>a_{12}/2\geq 0$ and have a normalization constant *N* expressed as:

$$N={\left(\frac{a_{1}+a_{1}^{*}-a_{12}}{\pi }\right)}^{1/2}\exp\left\{-\frac{{\left(b_{1}+b_{1}^{*}\right)}^{2}}{4(a_{1}+a_{1}^{*}-a_{12})}\right\}\;.$$

As discussed above, the Gaussian states can be fully identified by their covariance matrix and the mean values of the quadrature components. In the case of the state (7), the mean values of the operators $\hat{p}$ and $\hat{q}$ are:

(8) $$\langle \hat{p}\rangle \left(0\right)=b_{1I}-\frac{2a_{1I}b_{1R}}{2a_{1R}-a_{12}}\;,\;\langle \hat{q}\rangle \left(0\right)=\frac{b_{1R}}{2a_{1R}-a_{12}}\;,$$

and the initial covariance matrix of the system reads:

(9) $$\sigma \left(0\right)=\left(\begin{array}{cc} \sigma_{pp} & \sigma_{pq}\\ \sigma_{pq} & \sigma_{qq} \end{array}\right)=\frac{1}{2\;(2a_{1R}-a_{12})}\left(\begin{array}{cc} 4|a_{1}{|}^{2}-a_{12}^{2} & -2a_{1I}\\ -2a_{1I} & 1 \end{array}\right).$$

Here, the covariance between arbitrary operators $\hat{x}$ and $\hat{y}$ is given in terms of the expectation value of the anticommutator, i.e., $\sigma_{xy}=\frac{1}{2}\;\mathrm{Tr}\;\left(\hat{\rho }\left(\hat{x}\hat{y}+\hat{y}\hat{x}\right)\right)-\mathrm{Tr}\;\left(\hat{\rho }\hat{x}\right)\;\mathrm{Tr}\;\left(\hat{\rho }\hat{y}\right)$.

All properties of the Gaussian state can be obtained by the use of the covariance matrix and the mean values of the state. For example, the purity of the Gaussian state can be obtained by the determinant of its covariance matrix, that is,

(10) $$\mathrm{Tr}\;{\hat{\rho }}^{2}=\frac{1}{2\sqrt{\det\sigma }}\;.$$

The unitary dynamics of the initial state of Equation (7) can be obtained by the integration of the propagators multiplied by the mixed-state density matrix:

$$\rho \left(x,x^{\prime },t\right)=\int_{-\infty }^{\infty }\int_{-\infty }^{\infty }dx_{1}\;dx_{2}\;\;G^{*}\left(x_{1},x,t\right)\rho \left(x_{1},x_{2},0\right)\;G\left(x_{2},x^{\prime },t\right)\;;$$

as a result, it provides a Gaussian state with the same purity as the original state ($\mathrm{Tr}\;{\hat{\rho }}^{2}\left(t\right)=\mathrm{Tr}\;{\hat{\rho }}^{2}\left(0\right)$), since unitary transforms do not change purity, which then can infer the determinant invariance of the covariance matrix detσ(0)=detσ(t).

In a similar way, we can write the final state in an analogous way as the initial one, that is:

(11) $$\rho \left(x,x^{\prime },t\right)=N\exp\left\{-a_{1}\left(t\right)x^{2}+a_{12}\left(t\right)xx^{\prime }-a_{1}^{*}\left(t\right){x^{\prime }}^{2}+b\left(t\right)x+b{\left(t\right)}^{*}x^{\prime }\right\}\;,$$

where the Gaussian parameters are written in terms of the symplectic matrix associated with the invariants of Equation (5); thus, we arrive at the following expressions:

(12) $$\begin{array}{ccc} a_{1}\left(t\right) & = & \frac{1}{2\lambda_{4}}\left(\frac{2a_{1}^{*}\lambda_{4}-i\lambda_{1}}{\lambda_{1}^{2}-2i\left(a_{1}-a_{1}^{*}\right)\lambda_{1}\lambda_{4}+d\lambda_{4}^{2}}+i\lambda_{5}\right)\;,\\ a_{12}\left(t\right) & = & \frac{a_{12}}{\lambda_{1}^{2}-2i\left(a_{1}-a_{1}^{*}\right)\lambda_{1}\lambda_{4}+d\lambda_{4}^{2}}\;,\\ b(t) & = & \frac{\lambda_{1}\left(b-i\lambda_{3}+\left(a_{12}-2a_{1}\right)\lambda_{6}\right)+\lambda_{4}\left(\left(2a_{1}^{*}-a_{12}\right)\lambda_{3}+i\left(2a_{1}^{*}b+a_{12}b^{*}-\delta \lambda_{6}\right)\right)}{\lambda_{1}^{2}-2i\left(a_{1}-a_{1}^{*}\right)\lambda_{1}\lambda_{4}+d\lambda_{4}^{2}}, \end{array}$$

with $d=4|a_{1}{|}^{2}-a_{12}^{2}$.

It is possible to obtain the differential equations, which these parameters must satisfy. This is done by taking the time derivative of the parameters, in view of Equation (2); after some algebra, we obtain:

(13) $$\begin{array}{ccc} {\dot{a}}_{1}\left(t\right) & = & i\left(a_{12}^{2}\left(t\right)-4a_{1}^{2}\left(t\right)\right)\omega_{1}\left(t\right)-4a_{1}\left(t\right)\omega_{2}\left(t\right)+i\omega_{3}\left(t\right),\\ {\dot{a}}_{12}\left(t\right) & = & 4a_{12}\left(t\right)\left(i\left(a_{1}^{*}\left(t\right)-a_{1}\left(t\right)\right)\omega_{1}\left(t\right)-\omega_{2}\left(t\right)\right),\\ \dot{b}\left(t\right) & = & \left(2a_{1}\left(t\right)-a_{12}\left(t\right)\right)\delta_{1}\left(t\right)-i\delta_{2}\left(t\right)-2ia_{12}\left(t\right)b^{*}\left(t\right)\omega_{1}\left(t\right)-2b\left(t\right)\left(\omega_{2}\left(t\right)+2ia_{1}\left(t\right)\omega_{1}\left(t\right)\right)\;, \end{array}$$

and their corresponding complex conjugates. It is worth mentioning that these equations can be corroborated by the use of the von Neumann equation for $\rho (x,x^{\prime },t)$ given in Equation (11), namely $\;i\;\frac{d\rho (x,x^{\prime },t)}{dt}=\langle x|\left[\hat{H},\hat{\rho }\right]|x^{\prime }\rangle$. On the other hand, it is known that the covariance matrix of the system can be obtained, in view of the quantum solutions of Equation (2); as we have pointed out, this corresponds to the classical solutions (3) with $\delta_{1}=\delta_{2}=0$, which can be also written in terms of the symplectic transformation Λ of Equation (5), i.e., $\sigma \left(t\right)=\Lambda^{-1}\sigma \left(0\right){\tilde{\Lambda }}^{-1}$. Then, the covariance matrix evolution σ(t) can be obtained as:

(14) $$\sigma \left(t\right)=\left(\begin{array}{cc} \sigma_{pp}\left(t\right) & \sigma_{pq}\left(t\right)\\ \sigma_{pq}\left(t\right) & \sigma_{qq}\left(t\right) \end{array}\right)=\left(\begin{array}{cc} \lambda_{5} & -\lambda_{2}\\ -\lambda_{4} & \lambda_{1} \end{array}\right)\left(\begin{array}{cc} \sigma_{pp}\left(0\right) & \sigma_{pq}\left(0\right)\\ \sigma_{pq}\left(0\right) & \sigma_{qq}\left(0\right) \end{array}\right)\left(\begin{array}{cc} \lambda_{5} & -\lambda_{4}\\ -\lambda_{2} & \lambda_{1} \end{array}\right)\;.$$

After differentiating each covariance ${\dot{\sigma }}_{pp}\left(t\right)$, ${\dot{\sigma }}_{qq}\left(t\right)$, and ${\dot{\sigma }}_{pq}\left(t\right)$, by using Equation (2), the inverse expression of Equation (14), the purity conservation condition $\sigma_{pp}\left(0\right)\sigma_{qq}\left(0\right)-\sigma_{pq}^{2}\left(0\right)=\sigma_{pp}\left(t\right)\sigma_{qq}\left(t\right)-\sigma_{pq}^{2}\left(t\right)$, and the condition $\lambda_{1}\lambda_{5}-\lambda_{2}\lambda_{4}=1$, we arrive at the following differential equations for the covariances:

(15) $$\begin{array}{c} {\dot{\sigma }}_{pp}\left(t\right)=-4\;\left(\omega_{2}\left(t\right)\sigma_{pp}\left(t\right)+\omega_{3}\left(t\right)\sigma_{pq}\left(t\right)\right)\;,\\ {\dot{\sigma }}_{qq}\left(t\right)=4\;\left(\omega_{2}\left(t\right)\sigma_{qq}\left(t\right)+\omega_{1}\left(t\right)\sigma_{pq}\left(t\right)\right),\\ {\dot{\sigma }}_{pq}\left(t\right)=2\;\left(\omega_{1}\left(t\right)\sigma_{pp}\left(t\right)-\omega_{3}\left(t\right)\sigma_{qq}\left(t\right)\right)\;. \end{array}$$

One can also check that these differential equations imply that the derivative of the determinant of σ(t) is equal to zero, i.e., $\frac{d}{dt}\left[\sigma_{p}p\left(t\right)\sigma_{qq}\left(t\right)-\sigma_{pq}^{2}\right]=0$, which also implies that the purity of the state (11) is time invariant. It is noteworthy that the time-derivative expressions for the covariance matrix can be expressed as follows:

(16) $$\dot{\sigma }\left(t\right)=2\left[\sigma (t)B(t)\Sigma -\Sigma B(t)\sigma (t)\right]\;,$$

where the matrix B(t) contains the Hamiltonian coefficients, while Σ is a symplectic matrix, i.e.,

$$B\left(t\right)=\left(\begin{array}{cc} \omega_{1}\left(t\right) & \omega_{2}\left(t\right)\\ \omega_{2}\left(t\right) & \omega_{3}\left(t\right) \end{array}\right)\;,\;\Sigma =\left(\begin{array}{cc} 0 & 1\\ -1 & 0 \end{array}\right)\;.$$

On the other hand, one can also check, using the inverse expression of Equation (5), that the mean values of $\hat{p}$ and $\hat{q}$ follow the classical Equation (3), i.e.,

(17) $$\frac{d}{dt}\left(\begin{array}{c} \langle \hat{p}\rangle \\ \langle \hat{q}\rangle \end{array}\right)=2\left(\begin{array}{cc} -\omega_{2} & -\omega_{3}\\ \omega_{1} & \omega_{2} \end{array}\right)\left(\begin{array}{c} \langle \hat{p}\rangle \\ \langle \hat{q}\rangle \end{array}\right)+\left(\begin{array}{c} -\delta_{1}\\ \delta_{2} \end{array}\right)\;.$$

All information regarding the evolution of the Gaussian state can then be obtained by solving the differential Equations (16) and (17). As an example, we can consider the evolution of a Gaussian state with the initial covariance matrix σ(0) and mean values $\langle \hat{p}\rangle \left(0\right)$ and $\langle \hat{q}\rangle \left(0\right)$.

#### Example

As an example, we consider the following Hamiltonian:

(18) $$\hat{H}=\frac{1}{2}\left({\hat{p}}^{2}+\omega^{2}{\hat{q}}^{2}\right)+\frac{\nu }{2}\left(\hat{p}\hat{q}+\hat{q}\hat{p}\right)\;.$$

In view of Equations (16) and (17), the matrix B can be identified as:

$$B=\frac{1}{2}\left(\begin{array}{cc} 1 & \nu \\ \nu & \omega^{2} \end{array}\right)\;,$$

and one can show that, in the case of constant frequencies (ω, ν), the evolution is determined by the same differential equations for all the covariances:

$${\dddot{\sigma }}_{pp}=-4\left(\omega^{2}-\nu^{2}\right){\dot{\sigma }}_{pp}\;,\;{\dddot{\sigma }}_{qq}=-4\left(\omega^{2}-\nu^{2}\right){\dot{\sigma }}_{qq}\;,\;{\dddot{\sigma }}_{pq}=-4\left(\omega^{2}-\nu^{2}\right){\dot{\sigma }}_{pq}\;,$$

which at $\omega^{2}>\nu^{2}$ describes an oscillating motion with parameter $f^{2}=4\left(\omega^{2}-\nu^{2}\right)$. The solution to these equations, which satisfy the initial conditions for the derivatives and second derivatives at time t=0 implied by Equation (16), are:

(19) $$\begin{array}{c} \sigma_{pp}\left(t\right)=-\frac{2\left(f\sin\left(ft\right)\left(w^{2}z_{0}+\nu x_{0}\right)+\cos\left(ft\right)\left(\omega^{4}y_{0}-\omega^{2}\left(x_{0}-2\nu z_{0}\right)+2\nu^{2}x_{0}\right)-\omega^{2}\left(\omega^{2}y_{0}+x_{0}+2\nu z_{0}\right)\right)}{f^{2}}\;,\\ \sigma_{qq}\left(t\right)=\frac{2\left(-\cos\left(ft\right)\left(\omega^{2}\left(-y_{0}\right)+x_{0}+2\nu \left(\nu y_{0}+z_{0}\right)\right)+f\sin\left(ft\right)\left(\nu y_{0}+z_{0}\right)+\omega^{2}y_{0}+x_{0}+2\nu z_{0}\right)}{f^{2}}\;,\\ \sigma_{pq}\left(t\right)=\frac{2\cos\left(ft\right)\left(\omega^{2}\left(\nu y_{0}+2z_{0}\right)+\nu x_{0}\right)+f\sin\left(ft\right)\left(x_{0}-\omega^{2}y_{0}\right)-2\nu \left(\omega^{2}y_{0}+x_{0}+2\nu z_{0}\right)}{f^{2}}\;, \end{array}$$

with $x_{0}=\sigma_{pp}\left(0\right)$, $y_{0}=\sigma_{qq}\left(0\right)$, and $z_{0}=\sigma_{pq}\left(0\right)$. The solution for the classical equations of motion for the mean values reads:

(20) $$\begin{array}{ccc} \langle \hat{p}\rangle \left(t\right) & = & \langle \hat{p}\rangle \left(0\right)\cos\left(ft/2\right)-\left(2/f\right)\left[\omega^{2}\langle \hat{q}\rangle \left(0\right)+\nu \langle \hat{p}\rangle \left(0\right)\right]\sin\left(ft/2\right)\;,\\ \langle \hat{q}\rangle \left(t\right) & = & \langle \hat{q}\rangle \left(0\right)\cos\left(ft/2\right)+\left(2/f\right)\left[\nu \langle \hat{q}\rangle \left(0\right)+\langle \hat{p}\rangle \left(0\right)\right]\sin\left(ft/2\right)\;. \end{array}$$

**Figure 1:**
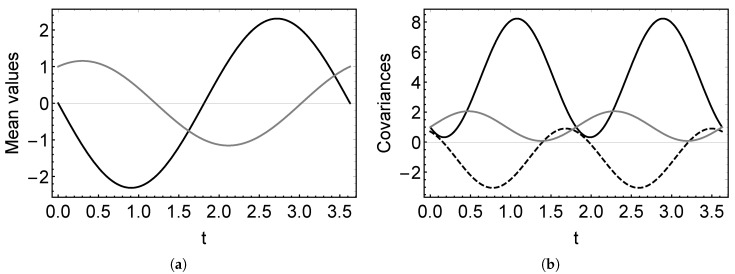
(**a**) Mean values $\langle \hat{p}\rangle \left(t\right)$ (black) and $\langle \hat{q}\rangle \left(t\right)$ (gray) for the dynamics of Hamiltonian (18) and the state with initial conditions $\langle \hat{p}\rangle \left(0\right)=0$ and $\langle \hat{q}\rangle \left(0\right)=1$. (**b**) Covariances $\sigma_{pp}\left(t\right)$ (black), $\sigma_{qq}\left(t\right)$ (gray), and $\sigma_{pq}\left(t\right)$ (dashed) for the initial state with $\sigma_{pp}\left(0\right)=\sigma_{qq}=1$ and $\sigma_{pq}\left(0\right)=1/\sqrt{2}$. In both cases, we took frequencies ω=2 and ν=1.

With these solutions, one can characterize the state behavior. In Figure 1, we show the evolution of the mean values and covariance matrix given by Equations (19) and (20) for the Hamiltonian (18). Here, we observe the oscillating behavior of the system. We would like to remark that, using these solutions for the covariances and the correspondence between the density matrix parameters and the covariances:

(21) $$\sigma \left(t\right)=\frac{1}{2(a_{1}\left(t\right)+a_{1}^{*}\left(t\right)-a_{12}\left(t\right))}\left(\begin{array}{cc} 4a_{1}\left(t\right)a_{1}^{*}\left(t\right)-a_{12}^{2}\left(t\right) & i(a_{1}\left(t\right)-a_{1}^{*}\left(t\right))\\ i(a_{1}\left(t\right)-a_{1}^{*}\left(t\right)) & 1 \end{array}\right),$$

one can also obtain the solution for the nonlinear Equation (13).

### 2.2. Invariant States

After obtaining the differential equations determining the evolution of the covariance matrix (16), one can ask the question: Do invariant states exist under the evolution of the quadratic Hamiltonian? To answer this question, we should examine the properties of Equation (16). If we assume the condition $\dot{\sigma }\left(t\right)=0$, then one needs to obtain all the covariance matrices, which satisfy the condition σ(t)B(t)Σ−ΣB(t)σ(t)=0. By taking the vector $\tilde{v}=\left(\sigma_{pp},\sigma_{pq},\sigma_{qq}\right)$, the equations for $\dot{\sigma }\left(t\right)=0$ can be written as follows:

(22) $$\mathrm{Mv}=0,\;\mathrm{with}\;M=\left(\begin{array}{ccc} -4\omega_{2} & -4\omega_{3} & 0\\ 2\omega_{1} & 0 & -2\omega_{3}\\ 0 & 4\omega_{1} & 4\omega_{2} \end{array}\right)\;.$$

As the matrix M has rank R=2, one can conclude that there is one nontrivial vector satisfying Equation (22). Exploring the null-space of M, one can check that the vector:

$$\tilde{v}=C\left(\frac{\omega_{3}}{\omega_{1}},-\frac{\omega_{2}}{\omega_{1}},1\right)\;,$$

with *C* being a constant, is the solution to Equation (22). We infer that all the states with a covariance matrix, given by:

(23) $$\sigma \left(0\right)=C\left(\begin{array}{cc} \frac{\omega_{3}}{\omega_{1}} & -\frac{\omega_{2}}{\omega_{1}}\\ -\frac{\omega_{2}}{\omega_{1}} & 1 \end{array}\right),$$

have an invariant covariance matrix. Using the inverse expressions of Equation (9), one can obtain an explicit form of the covariance invariant density matrix function. The parameters of the density operator (11) read:

(24) $$a_{1}=\frac{4C^{2}\omega_{1}\omega_{3}+{\left(\omega_{1}+2iC\omega_{2}\right)}^{2}}{8C\omega_{1}^{2}}\;,\;a_{12}=\frac{4S-1}{4C}\;,$$

with $S=C^{2}\left(\omega_{3}/\omega_{1}-\omega_{2}^{2}/\omega_{1}^{2}\right)$ being the determinant of the invariant covariance matrix. In the case of the Hamiltonian (18), we have $S=C^{2}\left(\omega^{2}-\nu^{2}\right)$, $\omega_{3}/\omega_{1}=\omega^{2}$, and $\omega_{2}/\omega_{1}=\nu$, which lead us to realize that any state with parameters, as in Equation (24) with S>1/4, is a bonafide quantum state, which is covariance invariant. For C=1, ω=2, and ν=1, we have that the states with:

(25) $$a_{1}=\frac{13}{8}+\frac{i}{2},\;a_{12}=\frac{11}{4}$$

are covariance invariant.

The states that satisfy $\dot{\sigma }=0$ or equivalently have parameters according to (24) are the states that do not change its shape on the phase space q,p; also, their mean values move following the classical equations of motion. If we assume these types of states with initial mean values $\langle \hat{p}\rangle \left(0\right)=\langle \hat{q}\rangle \left(0\right)=0$, the resulting states will be invariant, i.e., they will not change any of their properties over time (for $\delta_{1}=\delta_{2}=0$). An example of such states for the Hamiltonian (18) are the ones in Equation (11), with parameters given by (25) and $b\left(t\right)=b^{*}\left(t\right)=0$. We would like to express that, in the case of an invariant system with vanishing mean values, the initial energy will be different from zero as the initial covariances are also different from zero.

This parametric formalism for the evolution of Gaussian states and the definition of invariant states can be generalized to any multidimensional quadratic system as seen in the following section.

## 3. Multidimensional Quadratic System

In this section, we review the equations determining the evolution of the covariance matrix and mean values $\langle p_{j}\rangle$ and $\langle q_{j}\rangle$ for an arbitrary system under the evolution of a quadratic Hamiltonian; also, we mention the connection and dynamics of the continuous density matrix parameters. To obtain these properties, we use, as in the one-dimensional case, the invariant operators defined in [33,34].

In the case of an *N*-dimensional quadratic system, the time evolution is characterized by the Hamiltonian:

(26) $$\hat{H}=\tilde{r}B\left(t\right)r+\tilde{\Delta }\left(t\right)r\;,$$

where the tilde corresponds to the transposition operation and the vector $\tilde{r}=\left({\hat{p}}_{1},{\hat{q}}_{1},{\hat{p}}_{2},{\hat{q}}_{2},\ldots ,{\hat{p}}_{N},{\hat{q}}_{N}\right)$ corresponds to the vector of quadrature operators. The time dependence of this Hamiltonian is contained in the matrices:

(27) $$B\left(t\right)=\left(\begin{array}{cccc} \omega_{1,1}\left(t\right) & \omega_{1,2}\left(t\right) & \cdots & \omega_{1,2N}\left(t\right)\\ \omega_{1,2}\left(t\right) & \omega_{2,2}\left(t\right) & \cdots & \omega_{2,2N}\left(t\right)\\ \vdots & \vdots & \ddots & \vdots \\ \omega_{1,2N}\left(t\right) & \omega_{2,2N}\left(t\right) & \cdots & \omega_{2N,2N}\left(t\right) \end{array}\right),\;\Delta \left(t\right)=\left(\begin{array}{c} \delta_{1}\left(t\right)\\ \delta_{2}\left(t\right)\\ \vdots \\ \delta_{2N}\left(t\right) \end{array}\right)\;,$$

where B(t) is a real and symmetric matrix and Δ(t) is a real vector. As in the one-dimensional case, there exist 2N linear time-dependent operators ${\hat{R}}_{j}$ (j=1,…,2N), whose time derivatives are equal to zero $\left(\frac{d{\hat{R}}_{j}}{dt}=0.\right)$. These operators can be arranged on a vector as follows:

(28) R=Λ(t)r+Γ(t),

with the matrix Λ(t) and the vector $\tilde{\Gamma }\left(t\right)=\left(\gamma_{1}\left(t\right),\gamma_{2}\left(t\right),\ldots ,\gamma_{2N}\left(t\right)\right)$. By taking the time-derivative of the operator $R_{j}={\hat{R}}_{j}$ and equating it to zero $\left(\frac{d{\hat{R}}_{j}}{dt}=0.\right)$, one can demonstrate that Λ(t) and Γ(t) must satisfy the following differential equations:

(29) $$\dot{\Lambda }\left(t\right)=2i\Lambda \left(t\right)DB\left(t\right),\;\dot{\Gamma }\left(t\right)=i\Lambda \left(t\right)D\Delta \left(t\right),\;\mathrm{with}\;D_{j,k}=\left[r_{j},r_{k}\right]\;.$$

The solution to these differential equations with the initial conditions ${\hat{R}}_{j}\left(0\right)=r_{j}$ provides, as a result, the invariant operators $\tilde{R}=\left({\hat{P}}_{1},{\hat{Q}}_{1},{\hat{P}}_{2},{\hat{Q}}_{2},\ldots ,{\hat{P}}_{N},{\hat{Q}}_{N}\right)$, satisfying the standard commutation rules for the operators r at time equal to zero: i.e., $\left[R_{j},R_{k}\right]=\left[r_{j},r_{k}\right]=D_{j,k}$. This property leads us to the conclusion that the matrix Λ(t) must be symplectic and satisfies the equation:

(30) $$\Lambda \left(t\right)D\tilde{\Lambda }\left(t\right)=D.$$

This relation can be then used to obtain the inverse of Λ(t), which results in the expression:

(31) $$\Lambda^{-1}\left(t\right)=D\tilde{\Lambda }\left(t\right)D\;.$$

The other important property of these invariant operators is that they correspond to the inverse evolution of the original operators, in other words,

$$R_{j}=\hat{U}\left(t\right)r_{j}{\hat{U}}^{†}\left(t\right),$$

which in the most cases can be obtained from the Heisenberg picture operators by assuming a time reversal operation. This property implies that the entries of $\dot{\Lambda }\left(t\right)$ in Equation (29) satisfy the classical Hamilton equations after the time reversal operation, that is after the change $p_{i}\to -p_{i}$ in the classical Hamilton equations.

By the use of these invariant operators, one can obtain the time dependence of the mean values of the operators in r (〈r〉(t)) and their covariances $\sigma_{j,k}=\frac{1}{2}\langle \left\{r_{j},r_{k}\right\}\rangle -\langle r_{j}\rangle \langle r_{k}\rangle$. From the inverse of Equation (28), one can demonstrate that:

$$\langle r\rangle \left(t\right)=\Lambda^{-1}\left(t\right)\left(\langle R\rangle -\Gamma \left(t\right)\right)\;,$$

as the invariant operators in R have a time derivative equal to zero, and they are equal to the standard operators r at zero time, then one can conclude that:

(32) $$\langle r\rangle \left(t\right)=\Lambda^{-1}\left(t\right)\left(\langle r\rangle \left(0\right)-\Gamma \left(t\right)\right)\;.$$

From an analogous argument, one can see that the covariance matrix reads:

(33) $$\sigma \left(t\right)=\Lambda^{-1}\left(t\right)\sigma \left(0\right){\tilde{\Lambda }}^{-1}\left(t\right)\;.$$

Then, to obtain the expression for the time-derivative of the mean values 〈r〉(t) and the covariance matrix σ(t), we make use of Equations (29) and (31)–(33) and arrive to the expressions:

(34) $$\frac{d}{dt}\langle r\rangle \left(t\right)=-iD\left(2B\left(t\right)\langle r\rangle \left(t\right)+\Delta \left(t\right)\right)\;,\;\frac{d}{dt}\sigma \left(t\right)=2i\left(\sigma \left(t\right)B\left(t\right)D-DB\left(t\right)\sigma \left(t\right)\right)\;.$$

These differential equations, being first obtained in [31,32], are the generalization of the one-dimensional case discussed in the previous section. In our case, the nonlinear differential equations for the density matrix parameters can be obtained by explicit calculation of the covariances at time *t*. The resulting equations can then be solved by the substitution of the solution of Equation (34) or by direct integration.

To make the relation easier to see, we point out that the 2N×2N symplectic matrix D contains in its diagonal blocks made of the 2×2 symplectic matrix Σ, that is,

$$D=-i\left(\begin{array}{ccccc} \Sigma & 0 & 0 & \cdots & 0\\ 0 & \Sigma & 0 & \cdots & 0\\ \vdots & \ddots & \ddots & \ddots & \vdots \\ 0 & \cdots & 0 & \Sigma & 0\\ 0 & 0 & \cdots & 0 & \Sigma \end{array}\right)\;.$$

One can also notice that the differential equations for the entries of the covariance matrix are linear and can be expressed in the following matrix form:

(35) $$\frac{d}{dt}v=Mv\;,$$

where v is an N(2N+1)-dimensional vector, which contains all the independent covariances in the *N*-partite system, and the matrix M is a square matrix of the same dimension that contains the Hamiltonian coefficients.

## 4. Nonunitary Evolution for Gaussian Subsystems

Assume that the operators in the Hamiltonian of Equation (26) correspond to the ones of a multipartite system, where the position and momentum for the *j*th part are given by ${\hat{p}}_{j}$ and ${\hat{q}}_{j}$, respectively. Given this, one can see that the evolution of the complete system is unitary, but each one of its parts evolves in a nonunitary way due to the correlations between these parts. When the complete system is Gaussian, each one of its parts is also Gaussian. To show this property, lets assume that the *N*-partite system can be determined by the following density matrix at time t=0:

(36) $$\langle x|\hat{\rho }\left(0\right)|x^{\prime }\rangle =N\exp\left\{-\frac{1}{2}\tilde{y}\;A\;y+\tilde{b}\;y\right\},$$

where $\tilde{x}=\left(x_{1},x_{2},\ldots ,x_{N}\right)$, ${\tilde{x}}^{\prime }=\left(x_{1}^{\prime },x_{2}^{\prime },\ldots ,x_{N}^{\prime }\right)$, and $\tilde{y}=\left(x_{1},x_{2},\ldots ,x_{N},x_{1}^{\prime },x_{2}^{\prime },\ldots ,x_{N}^{\prime }\right)$ are real vectors. Furthermore, we define the vector $\tilde{b}=\left(b_{1},b_{2},\ldots ,b_{N},b_{1}^{*},b_{2}^{*},\ldots ,b_{N}^{*}\right)$ and the matrix:

$$A=\left(\begin{array}{cc} u & -v\\ -\tilde{v} & u^{*} \end{array}\right)\;,$$

where the block matrices u and v can be written as:

$$u=\left(\begin{array}{cccc} 2a_{1,1} & -a_{1,2} & \cdots & -a_{1,N}\\ -a_{1,2} & 2a_{2,2} & \cdots & -a_{2,N}\\ \vdots & \vdots & \ddots & \vdots \\ -a_{1,N} & -a_{2,N} & \cdots & 2a_{N,N} \end{array}\right)\;,\;v=\left(\begin{array}{ccccc} a_{1,N+1} & a_{1,N+2} & \cdots & a_{1,2N-1} & a_{1,2N}\\ a_{1,N+2}^{*} & a_{2,N+2} & \cdots & a_{2,2N-1} & a_{2,2N}\\ \vdots & \vdots & \ddots & \vdots & \vdots \\ a_{1,2N-1}^{*} & a_{2,2N-1}^{*} & \cdots & a_{N-1,2N-1} & a_{N-1,2N}\\ a_{1,2N}^{*} & a_{2,2N}^{*} & \cdots & a_{N,2N-1}^{*} & a_{N,2N} \end{array}\right)\;.$$

As stated earlier, the dynamics of the composite system is determined by the evolution of its covariance matrix and the mean values of the position and momentum operators, i.e., by the solution of Equation (34) with the initial state (36). The resulting state has the same purity as the initial state, since the evolution is unitary. However, there exists a nonunitary evolution of the parts, which compose the *N*-partite system.

To obtain the dynamic evolution of one of the parts, we can use the partial trace method. In other words, one should take the partial trace of all the subsystems in $\langle x|\hat{\rho }\left(t\right)|x^{\prime }\rangle$, except the one we want to study. Nevertheless, as the system is Gaussian, the partial traces should give us also a Gaussian state for the density matrix under study.

As the most general one-dimensional Gaussian state can be obtained by the 2×2 covariance matrix and the mean values ($\langle \hat{p}\rangle \left(t\right)$ and $\langle \hat{q}\rangle \left(t\right)$), we can obtain the result from the solutions to Equation (34) without the necessity of the partial trace operation.

On the other hand, once the time derivatives of these properties are established, one can derive the differential equation that the density matrix for the subsystem must satisfy. To show this procedure, we can take the bipartite system as an example.

### 4.1. Nonunitary Evolution on a Bipartite System

To exemplify the nonunitary evolution of a subsystem within a system, one can take a bipartite Gaussian state, which evolves on the Hamiltonian:

(37) $$\begin{array}{cc} \hat{H}\left(t\right)=\tilde{r}B\left(t\right)r+\tilde{\Gamma }\left(t\right)r & =\left({\hat{p}}_{1},{\hat{q}}_{1},{\hat{p}}_{2},{\hat{q}}_{2}\right)\left(\begin{array}{cccc} \omega_{1,1} & \omega_{1,2} & \omega_{1,3} & \omega_{1,4}\\ \omega_{1,2} & \omega_{2,2} & \omega_{2,3} & \omega_{2,4}\\ \omega_{1,3} & \omega_{2,3} & \omega_{3,3} & \omega_{3,4}\\ \omega_{1,4} & \omega_{2,4} & \omega_{3,4} & \omega_{4,4} \end{array}\right)\left(\begin{array}{c} {\hat{p}}_{1}\\ {\hat{q}}_{1}\\ {\hat{p}}_{2}\\ {\hat{q}}_{2} \end{array}\right)\\ \; & +\left(\gamma_{1},\gamma_{2},\gamma_{3},\gamma_{4}\right)\left(\begin{array}{c} {\hat{p}}_{1}\\ {\hat{q}}_{1}\\ {\hat{p}}_{2}\\ {\hat{q}}_{2} \end{array}\right), \end{array}$$

where $\omega_{j,k}$ and $\gamma_{j}$ may be functions of time. In order to determine the time evolution of the system, one can solve the differential equations defined for the covariance matrix and the mean values of the position and momentum operators or, similarly to the one-dimensional case, one can solve the equations for the density matrix parameters given in Equation (A3) of Appendix A. The differential equations for the covariance matrix and mean values of the position and momentum operators for the subsystem can be obtained using Equation (34). To solve the time derivative equations, we express the matrices B(t), D, and σ(t) in the 2×2 block representation; in such a case, we have:

$$B\left(t\right)=\left(\begin{array}{cc} B_{1}\left(t\right) & B_{1,2}\left(t\right)\\ {\tilde{B}}_{1,2}\left(t\right) & B_{2}\left(t\right) \end{array}\right)\;,\;D=\left(\begin{array}{cc} -i\Sigma & 0\\ 0 & -i\Sigma \end{array}\right)\;,\;\sigma \left(t\right)=\left(\begin{array}{cc} \sigma_{1}\left(t\right) & \sigma_{1,2}\left(t\right)\\ {\tilde{\sigma }}_{1,2}\left(t\right) & \sigma_{2}\left(t\right) \end{array}\right)\;,$$

where $\sigma_{1}\left(t\right)$ and $\sigma_{2}\left(t\right)$ are the covariance matrices for Subsystems 1 and 2, respectively, and $\sigma_{1,2}\left(t\right)$ is a matrix containing the covariances associated with the correlations between the two subsystems. The same can be said for the matrix linked to the Hamiltonian (37), i.e., B(t) where the block matrices $B_{1}\left(t\right)$ and $B_{2}\left(t\right)$ are associated with Subsystems 1 and 2, respectively, while $B_{1,2}$ is associated with the interactions between these two subsystems.

After this identification, the expression for the covariance matrices of the subsystems and the correlations can be given as follows:

(38) $$\begin{array}{c} {\dot{\sigma }}_{1}\left(t\right)=2\left(\left(\sigma_{1}\left(t\right)B_{1}\left(t\right)+\sigma_{1,2}\left(t\right){\tilde{B}}_{1,2}\right)\Sigma -\Sigma \left(B_{1}\left(t\right)\sigma_{1}\left(t\right)+B_{1,2}{\tilde{\sigma }}_{1,2}\left(t\right)\right)\right)\;,\\ {\dot{\sigma }}_{2}\left(t\right)=2\left(\left(\sigma_{2}\left(t\right)B_{2}\left(t\right)+{\tilde{\sigma }}_{1,2}\left(t\right)B_{1,2}\right)\Sigma -\Sigma \left(B_{2}\left(t\right)\sigma_{2}\left(t\right)+{\tilde{B}}_{1,2}\sigma_{1,2}\left(t\right)\right)\right)\;,\\ {\dot{\sigma }}_{1,2}\left(t\right)=2\left(\left(\sigma_{1}\left(t\right)B_{1,2}\left(t\right)+\sigma_{1,2}\left(t\right)B_{2}\right)\Sigma -\Sigma \left(B_{1}\left(t\right)\sigma_{1,2}\left(t\right)+B_{1,2}\sigma_{2}\left(t\right)\right)\right)\;. \end{array}$$

Then, one can recognize the term $2(\sigma_{j}B_{j}\Sigma -\Sigma B_{j}\sigma_{j})$ for j=1,2, as the term corresponding to a unitary evolution of each subsystem (16). The extra term $2(\sigma_{1,2}{\tilde{B}}_{1,2}\Sigma -\Sigma B_{1,2}{\tilde{\sigma }}_{1,2})$ is associated with the nonunitary evolution of the subsystems.

It is worth noting that these results are in accordance with the ones described by Sandulescu et al. [36] and Isar [37,38], where those results were obtained by solving the Gorini–Kossakowski–Sudarshan–Lindblad master equation [28,29,30] for two coupled oscillators. The main difference here is that our results were obtained exactly from the von Neumann equation without introducing a master equation.

### 4.2. Invariant and Quasi-Invariant States

The expression for the derivatives of the covariance matrix can lead to the definition of different Gaussian states, which do not evolve in the Hamiltonian dynamics. These types of states can be found as solutions to the equation $\dot{\sigma }=0$, which can be expressed in terms of the following equation regarding the covariance and the Hamiltonian matrices σB(t)D−DB(t)σ=0. As discussed before, this system of differential equations can be replaced by $\dot{v}=\mathrm{Mv}$ (35) with the following correspondences:

$$\tilde{v}=\left(\sigma_{p_{1}p_{1}},\sigma_{p_{1}q_{1}},\sigma_{p_{1}p_{2}},\sigma_{p_{1}q_{2}},\sigma_{q_{1}q_{1}},\sigma_{q_{1}p_{2}},\sigma_{q_{1}q_{2}},\sigma_{p_{2}p_{2}},\sigma_{p_{2}q_{2}},\sigma_{q_{2}q_{2}}\right)\;,$$

and the matrix M containing the Hamiltonian coefficients is presented in Equation (A4) of Appendix B. It is possible to see that the matrix M has a determinant detM=0 and a rank R=8. From these properties, one can see that the system $\dot{\sigma }\left(t\right)=\dot{v}=0$ has at most two different nontrivial solutions, which may be physical.

To exemplify the definition of bipartite states, which have a stationary behavior, we consider the frequency converter and the parametric amplifier. Both of these systems are quadratic and model the interaction between different electromagnetic fields in a nonlinear medium.

### 4.3. Frequency Converter

The quantum frequency converter is a device where two different unimodal electromagnetic fields, called the input and the output, interact with a semiclassical pump field on a nonlinear material. This interaction has the goal of interchanging the frequencies of the input and output beams at specific times. This behavior can be modeled using the following Hamiltonian:

(39) $$\hat{H}\left(t\right)=\hbar \omega_{1}\left({\hat{a}}_{1}^{†}{\hat{a}}_{1}+1/2\right)+\hbar \omega_{2}\left({\hat{a}}_{2}^{†}{\hat{a}}_{2}+1/2\right)-\hbar \kappa \left({\hat{a}}_{1}^{†}{\hat{a}}_{2}e^{-i\omega t}+{\hat{a}}_{1}{\hat{a}}_{2}^{†}e^{i\omega t}\right)\;,$$

where the frequencies $\omega_{1,2}$ are the input and output frequencies, respectively, ω is the pump field frequency, and the bosonic operators ${\hat{a}}_{1,2}$ are the annihilation operators of the input and output fields, respectively. These beams interact with an intensity κ in a nonlinear medium as, e.g., a nonlinear crystal. In this case, the Hamiltonian matrix B(t) from (26) (in a unit system where ℏ=1) reads:

$$B\left(t\right)=\frac{1}{2}\left(\begin{array}{cccc} 1 & 0 & -\frac{\kappa }{\sqrt{\omega_{1}\omega_{2}}}\cos\left(\omega t\right) & \kappa \sqrt{\omega_{2}/\omega_{1}}\sin\left(\omega t\right)\\ 0 & \omega_{1}^{2} & -\kappa \sqrt{\omega_{1}/\omega_{2}}\sin\left(\omega t\right) & -\kappa \sqrt{\omega_{1}\omega_{2}}\cos\left(\omega t\right)\\ -\frac{\kappa }{\sqrt{\omega_{1}\omega_{2}}}\cos\left(\omega t\right) & -\kappa \sqrt{\omega_{1}/\omega_{2}}\sin\left(\omega t\right) & 1 & 0\\ \kappa \sqrt{\omega_{2}/\omega_{1}}\sin\left(\omega t\right) & -\kappa \sqrt{\omega_{1}\omega_{2}}\cos\left(\omega t\right) & 0 & \omega_{2}^{2} \end{array}\right)\;.$$

For this Hamiltonian, one can obtain different states that have dynamical equilibrium properties, i.e., states with a time derivative for the covariance matrix equal to zero. To characterize these types of states, one should solve Equation (34) with $\dot{\sigma }\left(t\right)=0$. As previously discussed, $\dot{\sigma }\left(t\right)=0$ can be expressed in vector form as:

(40) Mv=0,

where v is defined as:

$$v=\left(\sigma_{p_{1}p_{1}},\sigma_{p_{1}q_{1}},\sigma_{p_{1}p_{2}},\sigma_{p_{1}q_{2}},\sigma_{q_{1}q_{1}},\sigma_{q_{1}p_{2}},\sigma_{q_{1}q_{2}},\sigma_{p_{2}p_{2}},\sigma_{p_{2}q_{2}},\sigma_{q_{2}q_{2}}\right)\;,$$

and the matrix M is a 10×10 matrix of rank R=8, which contains the Hamiltonian parameters of B(t). To solve Equation (40), one can explore the null space of matrix M. The resulting null space contains two different vectors; one contains a non-physical solution. In this solution,

$$\begin{array}{ccc} \sigma_{p_{1}p_{1}}={\left(\omega_{1}^{3}\omega_{2}\right)}^{1/2}\left(\omega_{2}-\omega_{1}\right)\sec\left(\omega t\right)/\kappa , & \; & \sigma_{p_{1}p_{2}}=\omega_{1}\omega_{2},\\ \sigma_{p_{1}q_{2}}=-\omega_{1}\tan\left(\omega t\right), & \; & \sigma_{q_{1}q_{1}}=\omega_{2}^{1/2}\left(\omega_{2}-\omega_{1}\right)\sec\left(\omega t\right)/\left(\kappa \omega_{1}^{1/2}\right),\\ \sigma_{q_{1}p_{2}}=\omega_{2}\tan\left(\omega t\right), & \; & \sigma_{q_{1}q_{2}}=1, \end{array}$$

while all the other covariances are equal to zero. In particular, it contains the nonphysical terms $\sigma_{p_{2}p_{2}}=\sigma_{q_{2}q_{2}}=0$ that resemble the case where one of the subsystem is classical, as in a classical system, the values of the covariances can be equal to zero. The null space also contains another vector, which has the following physical values:

(41) $$\sigma_{p_{1}p_{1}}=\omega_{1}\omega_{2},\;\sigma_{q_{1}q_{1}}=\omega_{2}/\omega_{1},\;\sigma_{p_{2}p_{2}}=\omega_{2}^{2},\;\sigma_{q_{2}q_{2}}=1\;,$$

with the remaining covariances equal to zero.

These results led us to the conclusion that a two-mode Gaussian state with initial covariances proportional to the ones established in (41) has the same covariances for any time t>0 (for time-independent parameters ω, $\omega_{1,2}$, and κ). This property has several physical implications such as, for example, that the purity of the subsystems will always be the same regardless of the interaction between them and despite the interchange of their frequencies. The resulting states will only have different mean values of the quadrature components ($p_{1}$, $q_{1}$, $p_{2}$, and $q_{2}$), which evolve according to the classical Hamilton equations.

In the case where the mean values of the quadrature components $b_{j}$; j=1,2 are equal to zero in Equation (36), one can obtain different states, which do not change their properties over time. In this case, the entanglement of the system (which can be obtained by the logarithmic negativity of the covariance matrix) will also be an invariant of the system. These properties make the evolution of these types of states relevant to quantum computing and quantum information.

By the use of the inverse relations of Equations of the Appendix A: (A1) and (A2), one can then write a general state with an invariant covariance matrix, which only changes its mean values according to the classical motion equations. Such a state can be expressed as the one in (36), after making the identification:

(42) $$\begin{array}{c} A=\left(\begin{array}{cccc} \frac{\omega_{1}}{4C\omega_{2}}+C\omega_{1}\omega_{2} & 0 & \frac{\omega_{1}}{4C\omega_{2}}-C\omega_{1}\omega_{2} & 0\\ 0 & \frac{1}{4C}+C\omega_{2}^{2} & 0 & \frac{1}{4C}-C\omega_{2}^{2}\\ \frac{\omega_{1}}{4C\omega_{2}}-C\omega_{1}\omega_{2} & 0 & \frac{\omega_{1}}{4C\omega_{2}}+C\omega_{1}\omega_{2}\\ 0 & \frac{1}{4C}-C\omega_{2}^{2} & 0 & \frac{1}{4C}+C\omega_{2}^{2} \end{array}\right)\;, \end{array}$$

with *C* being a constant, which needs to be chosen in order for the covariance matrix to be positive; in particular, to fulfill the Schrödinger–Robertson inequalities $\sigma_{p_{i}p_{i}}\sigma_{q_{i}q_{i}}-\sigma_{p_{i}q_{i}}^{2}\geq 1/4$ (i=1,2) and detσ≥1/16.

### 4.4. Parametric Amplifier

The other Hamiltonian, which can be taken as an example, is the nondegenerate parametric amplifier. This system also describes the interaction of the input and output beams with the pump field in a nonlinear medium. As a result of this interaction, one can obtain the amplification of the input beam. The Hamiltonian associated with the parametric amplifier reads:

(43) $$\hat{H}=\hbar \omega_{1}\left({\hat{a}}_{1}^{†}{\hat{a}}_{1}+1/2\right)+\hbar \omega_{2}\left({\hat{a}}_{2}^{†}{\hat{a}}_{2}+1/2\right)-\hbar \kappa \left({\hat{a}}_{1}^{†}{\hat{a}}_{2}^{†}e^{-i\omega t}+{\hat{a}}_{1}{\hat{a}}_{2}e^{i\omega t}\right)\;,$$

where the frequencies $\omega_{1,2}$ are the frequencies of the input and output beam channels and ω is the frequency of a pump field, which allows the amplification of the input channel. Then, the Hamiltonian matrix B(t) is:

(44) $$B\left(t\right)=\frac{1}{2}\left(\begin{array}{cccc} 1 & 0 & \kappa \frac{\cos(\omega t)}{\sqrt{\omega_{1}\omega_{2}}} & \kappa \sqrt{\omega_{2}/\omega_{1}}\sin\left(\omega t\right)\\ 0 & \omega_{1}^{2} & \kappa \sqrt{\omega_{1}/\omega_{2}}\sin\left(\omega t\right) & -\kappa \sqrt{\omega_{1}\omega_{2}}\cos\left(\omega t\right)\\ \kappa \frac{\cos(\omega t)}{\sqrt{\omega_{1}\omega_{2}}} & \kappa \sqrt{\omega_{1}/\omega_{2}}\sin\left(\omega t\right) & 1 & 0\\ \kappa \sqrt{\omega_{2}/\omega_{1}}\sin\left(\omega t\right) & -\kappa \sqrt{\omega_{1}\omega_{2}}\cos\left(\omega t\right) & 0 & \omega_{2}^{2} \end{array}\right)\;.$$

Following an analogous procedure to obtain the solutions of the equation $\dot{\sigma }=0$, one can show that the null space of the corresponding matrix M for this problem can lead us to nonphysical values for different covariances on the system. One of the vectors of the null space for the case $\omega_{1,2}>0$ can be written as:

$$\begin{array}{ccc} \sigma_{p_{1}p_{1}} & = & \frac{\omega_{1}\sqrt{\omega_{1}\omega_{2}}\left(\omega_{1}+\omega_{2}\right)\sec\left(\omega t\right)}{\kappa },\;\sigma_{p_{1}p_{2}}=-\omega_{1}\omega_{2},\;\sigma_{p_{1}q_{2}}=-\omega_{1}\tan\left(\omega t\right),\\ \sigma_{q_{1}q_{1}} & = & \frac{\omega_{2}\left(\omega_{1}+\omega_{2}\right)\sec\left(\omega t\right)}{\kappa \sqrt{\omega_{1}\omega_{2}}},\;\sigma_{p_{2}q_{1}}=-\omega_{2}\tan\left(\omega t\right),\;\sigma_{q_{1}q_{2}}=1, \end{array}$$

while all the other covariances are equal to zero. The other vector on the null space is:

$$\sigma_{p_{1}p_{1}}=-\omega_{1}\omega_{2},\;\sigma_{q_{1}q_{1}}=-\frac{\omega_{2}}{\omega_{1}},\;\sigma_{p_{2}p_{2}}=\omega_{2}^{2},\;\sigma_{q_{2}q_{2}}=1.$$

As the condition $\omega_{1,2}>0$ was used to obtain these results, then both vectors lead to nonphysical covariances. Nevertheless, one can obtain states with a slow change ratio of the covariances compared with the change of the mean value of the system Hamiltonian $\langle \hat{H}\rangle \left(t\right)$. These type of states can be defined by considering the initial covariances equal to $C=1/(\omega_{1}\omega_{2})$ times the ones presented in Equation (41); in other words,

$$\sigma_{p_{1},p_{1}}=1,\;\sigma_{q_{1},q_{1}}=1/\omega_{1}^{2},\;\sigma_{p_{2},p_{2}}=\omega_{2}/\omega_{1},\;\sigma_{q_{2},q_{2}}=1/\left(\omega_{1}\omega_{2}\right)\;.$$

**Figure 2:**
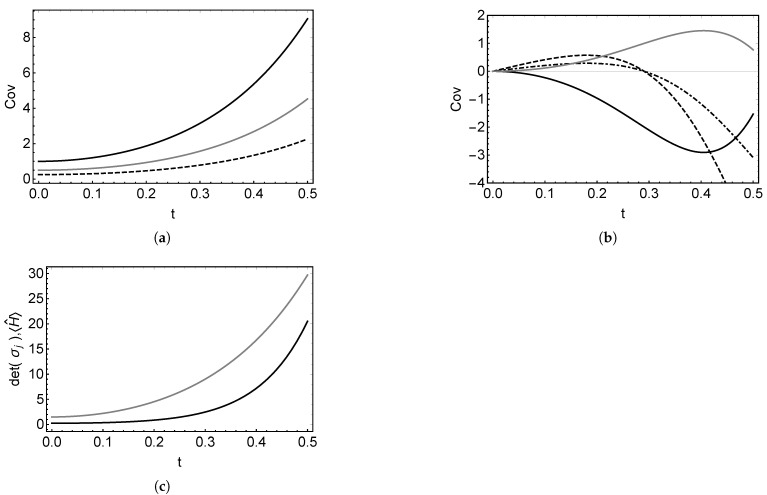
Time evolution for the covariances (**a**) $\sigma_{p_{1}p_{1}}$ (black), $\sigma_{q_{1}q_{1}}$ (dashed), and $\sigma_{p_{2}p_{2}}=\sigma_{q_{2}q_{2}}$ (gray) and (**b**) the covariances $\sigma_{p_{1}p_{2}}$ (black), $\sigma_{p_{1}q_{2}}$ (black dashed), $\sigma_{p_{2}q_{1}}$ (black dot-dashed), and $\sigma_{q_{1}q_{2}}$ (gray), (**c**) $\det\sigma_{1}=\det\sigma_{2}$ for the subsystems (black) and the time dependence of the mean value $\langle \hat{H}\rangle \left(t\right)$ (gray). For all the plots, the initial values are $\sigma_{p_{1}p_{1}}\left(0\right)=1$, $\sigma_{q_{1}q_{1}}\left(0\right)=1/4$, and $\sigma_{p_{2}p_{2}}\left(0\right)=\sigma_{q_{2}q_{2}}\left(0\right)=1/2$. All the other initial covariances are equal to zero. The frequencies used are $\omega_{1}=2$, $\omega_{2}=1$, ω=7, and $\kappa =\sqrt{10}$.

The slow time dependence behavior of these covariances can be seen in Figure 2, where the time dependence of the covariances and the purity of the subsystems are illustrated. The evolution of the subsystems in the parametric amplifier normally varies very fast, as the photons from the pump field are transformed into the photons of both subsystems. Nevertheless, it can be seen in Figure 2 that the variation of the majority of the covariances is not as fast compared with the change of $\langle \hat{H}\rangle \left(t\right)$, providing the strong coupling between the subsystems. In this particular example, one can see that $\sigma_{p_{2}p_{2}}\left(t\right)=\sigma_{q_{2}q_{2}}\left(t\right)$ and $\sigma_{p_{1}q_{1}}\left(t\right)=\sigma_{p_{2}q_{2}}\left(t\right)=0$.

The detection of these type of states can be done by the use of quantum tomography, as is discussed in the next section.

## 5. Gaussian States and Their Evolution in the Tomographic-Probability Representation

There exist different representations of quantum states [39,40,41,42,43,44], and among them, the probability tomographic representation is of particular interest. In this representation, e.g., one-mode photon states are identified with symplectic tomograms [45], which correspond to the conditional probability distribution w(X∣μ,ν) of the photon quadrature −∞<X<∞, to be measured in a reference frame with parameters μ=scosθ and $\nu =s^{-1}\sin\theta$. Here, −∞<μ,ν<∞, *s* is a time scaling parameter, and θ is the local oscillator phase, which is used in experiments [46] to obtain the Wigner function of the photon state.

The symplectic tomogram $w_{\rho }\left(X|\mu ,\nu \right)$ is determined by the photon density operator $\hat{\rho }$ [45] as:

(45) $$w_{\rho }\left(X|\mu ,\nu \right)=Tr\left[\hat{\rho }\;\delta \left(X\hat{1}-\mu \hat{q}-\nu \hat{p}\right)\right],$$

where $\hat{q}$ and $\hat{p}$ are quadrature components—the position and momentum operators within the framework of the oscillator model of the one-mode electromagnetic-field photons. The symplectic tomogram satisfies the normalization condition:

(46) $$\int w_{\rho }\left(X|\mu ,\nu \right)\;dX=1,$$

and inversely, it determines the density operator $\hat{\rho }$ of the photon state:

(47) $$\hat{\rho }=\frac{1}{2\pi }\int w\left(X|\mu ,\nu \right)\;\exp\left[i\;\delta (X\hat{1}-\mu \hat{q}-\nu \hat{p})\right]dX\;d\mu \;d\nu .$$

The optical tomogram of the photon state $w_{\mathrm{opt}}\left(X|\theta \right)\equiv w\left(X|\mu =\cos\theta ,\nu =\sin\theta \right)$ is measured in experiments, and in view of the homogeneity property of the Dirac delta-function, the measured optical tomogram of the photon state determines the symplectic tomogram:

(48) $$w\left(X|\mu ,\nu \right)=\frac{1}{\sqrt{\mu^{2}+\nu^{2}}}\;w_{\mathrm{opt}}\left[\frac{X}{\sqrt{\mu^{2}+\nu^{2}}}|\arctan\frac{\nu }{\mu }\right].$$

For Gaussian states (7), the tomographic-probability distribution of random photon quadrature *X* has the conventional form of a normal distribution:

(49) $$w\left(X|\mu ,\nu \right)=\frac{1}{\sqrt{2\pi \sigma (\mu ,\nu )}}\;\exp\left[-\frac{{\left(X-\bar{X}\left(\mu ,\nu \right)\right)}^{2}}{2\sigma (\mu ,\nu )}\right].$$

In view of (45), one has the mean value of the quadrature:

(50) $$\bar{X}\left(\mu ,\nu \right)=\mu \langle \hat{q}\rangle +\nu \langle \hat{p}\rangle$$

and the covariance of the quadrature σ(μ,ν) reads:

(51) $$\sigma \left(\mu ,\nu \right)=\mu^{2}\sigma_{qq}+\nu^{2}\sigma_{pp}+2\mu \nu \sigma_{pq}.$$

For the measured optical tomogram, the dispersion σ(θ) is:

(52) $$\sigma \left(\theta \right)=\left(\cos^{2}\theta \right)\sigma_{qq}+\left(\sin^{2}\theta \right)\sigma_{pp}+\left(\sin2\theta \right)\sigma_{pq}.$$

In the quantum system with Hamiltonian (18), the tomographic quadrature dispersion (51) evolves according to the formula:

(53) $$\sigma \left(\mu ,\nu ,t\right)=\mu^{2}\sigma_{qq}\left(t\right)+\nu^{2}\sigma_{pp}\left(t\right)+2\mu \nu \sigma_{pq}\left(t\right),$$

where $\sigma_{qq}\left(t\right)$, $\sigma_{pp}\left(t\right)$, and $\sigma_{pq}\left(t\right)$ are provided by the explicit expressions (19) and the parameters $\langle \hat{p}\left(t\right)\rangle$ and $\langle \hat{q}\left(t\right)\rangle$ are given by (20). Thus, the properties of the Gaussian states of the oscillator with time-dependent parameters described by the covariances of the position and momentum and their mean values can be checked by considering the covariance of the homodyne quadrature *X*, as well as the mean value evolution.

The properties of the invariant Gaussian states can be visualized by the properties of either the Wigner function or the tomographic-probability distributions. There are examples of the time-dependent Gaussian-packet solutions of the kinetic equation for the symplectic tomogram [47,48] in the case of the harmonic oscillator Hamiltonian (18) with ν=0. Since the dispersion matrix for the quadrature *X* is the linear combination of covariances $\sigma_{qq}\left(t\right)$, $\sigma_{pp}\left(t\right)$, and $\sigma_{pq}\left(t\right)$, which in the case of invariant Gaussian states, do not depend on time, the state tomogram also does not depend on time. The invariant states with density operators $|E_{n}\rangle \langle E_{n}|$ have the oscillator tomograms obtained from energy states $|E_{n}\rangle$, where $\hat{H}|E_{n}\rangle =E_{n}|E_{n}\rangle$. Tomograms of invariant Gaussian states satisfy the equality:

(54) $$P_{G}^{n}=\frac{1}{2\pi }\int w_{G}\left(X|\mu ,\nu \right)\;w_{E_{n}}\left(Y|\mu ,\nu \right)\;e^{i(X+Y)}\;dX\;dY\;d\mu \;d\nu ,$$

where the parameter $P_{G}^{n}$ is the probability to detect the properties of the stationary state $|E_{n}\rangle$ with energy value $E_{n}$ in the Gaussian state with the time-dependent tomogram $w_{G}\left(X|\mu ,\nu \right)$. This state also does not depend on time.

Any convex sum of states $|E_{n}\rangle \langle E_{n}|$ is a density operator. One can conjecture that there is the decomposition of normal distribution $w_{G}\left(X|\mu ,\nu \right)$ corresponding, e.g., to a thermal state with $\hat{\rho }=\exp\left(-\hat{H}/\left(kT\right)\right)/\mathrm{Tr}\left(\exp\left(-\hat{H}/\left(kT\right)\right)\right)$ (*T* being the temperature and *k* the Boltzmann constant), which can be presented as:

(55) $$w_{G}\left(X|\mu ,\nu \right)=\sum_{n}P_{G}^{n}\;w_{E_{n}}\left(X|\mu ,\nu \right),\;\sum_{n}P_{G}^{n}=1.$$

An analogous relation can be then written also for the Wigner function of the invariant Gaussian state of the oscillator, as well as for the density matrix in the position representation.

Now, we consider a harmonic oscillator with the Hamiltonian $\hat{H}=\frac{{\hat{p}}^{2}}{2}+\frac{{\hat{q}}^{2}}{2}$. The density matrix of the thermal equilibrium state with temperature $T=\beta^{-1}$ in the position representation reads (here, we assume ℏ=ω=m=k=1):

(56) $$\rho \left(x,x^{\prime },\beta \right)={\left[\pi^{-1}\tan^{2}\left(\beta /2\right)\right]}^{1/2}\exp\left[\frac{xx^{\prime }}{\sinh\beta }-\frac{x^{2}+{x^{\prime }}^{2}}{2}\coth\beta \right].$$

The Green function of the oscillator has the Gaussian form:

(57) $$G\left(x,x^{\prime },t\right)=\langle x|e^{-it\hat{H}}|x^{\prime }\rangle =\frac{1}{\sqrt{2\pi i\sin t}}\exp\left[\frac{i(x^{2}+{x^{\prime }}^{2})}{2}\cot t-\frac{ixx^{\prime }}{\sin t}\right].$$

Since the density matrix (56) is determined by the Green function (57), i.e.,

(58) $$\rho \left(x,x^{\prime },\beta \right)=\frac{G(x,x^{\prime },-i\beta )}{Z(\beta )},$$

with the partition function Z(β) given by the formula:

(59) $$Z\left(\beta \right)=\sum_{n=0}^{\infty }\mathrm{Tr}\left[\exp\left(-\beta \hat{H}\right)|n\rangle \langle n|\right]=\frac{1}{2\sinh(\beta /2)}\;;$$

here, we use the property $\hat{H}|n\rangle =\left(n+1/2\right)|n\rangle$. The density matrix (56) does not depend on time; this means that in all other representations, as the Wigner function or tomographic-probability representation, it is time-invariant. The density matrices of Fock states ∣n〉〈n∣ do not depend on time.

In the position representation, the density matrix of Fock state ∣n〉〈n∣ reads:

(60) $$\langle x|n\rangle \langle n|x^{\prime }\rangle =\frac{H_{n}\left(x\right)H_{n}\left(x^{\prime }\right)}{2^{n}n!\sqrt{\pi }}\;\exp\left(-\frac{x^{2}}{2}-\frac{x^{\prime 2}}{2}\right),$$

and it does not depend on time.

The density matrix (56), being described by an invariant Gaussian function, is the convex sum of the Fock state density matrices. One has the relation:

(61) $$\rho \left(x,x^{\prime },\beta \right)=\frac{1}{\sqrt{\pi }}\;\exp\left(-\frac{x^{2}}{2}-\frac{x^{\prime 2}}{2}\right)\sum_{n=0}^{\infty }\frac{e^{-(n+1/2)\beta }}{Z\left(\beta \right)2^{n}n!}\;H_{n}\left(x\right)H_{n}\left(x^{\prime }\right),$$

where Z(β) is given in (59).

In the tomographic-probability representation of the thermal equilibrium oscillator and Fock states, we can calculate the tomograms in explicit form. For Fock states,

(62) $$w_{n}\left(X|\mu ,\nu ,\beta \right)={\left[\pi (\mu^{2}+\nu^{2})\right]}^{-1/2}\frac{1}{2^{n}n!}\;\exp\left(-\frac{X^{2}}{\mu^{2}+\nu^{2}}\right)\;H_{n}^{2}\left(\frac{X}{\sqrt{\mu^{2}+\nu^{2}}}\right).$$

With all these properties, one can check that the thermal equilibrium Gaussian state of Equation (58) has a symplectic tomogram in the form of the normal distribution:

(63) $$w\left(X|\mu ,\nu ,\beta \right)=\frac{1}{\sqrt{2\pi \sigma (\mu ,\nu )}}\;\exp\left(-\frac{X^{2}}{2\sigma (\mu ,\nu )}\right).$$

The dispersion of quadrature $\langle X^{2}\rangle =\sigma \left(\mu ,\nu \right)$ given by Equation (63) reads:

(64) $$\sigma \left(\mu ,\nu \right)=\mu^{2}\langle {\hat{q}}^{2}\rangle +\nu^{2}\langle {\hat{p}}^{2}\rangle ,$$

where the state with density matrix (56):

(65) $$\langle {\hat{q}}^{2}\rangle =\langle {\hat{p}}^{2}\rangle =\frac{1}{2}\;\coth^{2}\left(\beta /2\right).$$

Thus, the symplectic tomogram (63) is given by an invariant normal probability distribution:

(66) $$w\left(X|\mu ,\nu ,\beta \right)=\frac{\coth(\beta /2)}{\sqrt{\pi (\mu^{2}+\nu^{2})}}\;\exp\left(-\frac{X^{2}}{\mu^{2}+\nu^{2}}\;\coth^{2}\left(\beta /2\right)\right).$$

Since for optical tomogram μ=cosθ, ν=sinθ, and $\mu^{2}+\nu^{2}=1$, in the case of the thermal single-mode photon state, its optical tomogram is:

(67) $$w\left(X|\theta ,\beta \right)=\frac{\coth(\beta /2)}{\sqrt{\pi }}\;\exp\left(-X^{2}\;\coth^{2}\left(\beta /2\right)\right);$$

it depends neither on the local oscillator phase, nor on time. These types of states are Gaussian and time-independent, so there is a connection between them and the invariant states discussed above. This connection can be checked by equaling the covariance matrices in both cases, which can be also checked experimentally, for example, by preparing an initial Gaussian state according to the invariance condition $\dot{\sigma }=0$. As seen in previous examples, this condition implies a value for the initial covariances in terms of the Hamiltonian parameters. Then, using the tomographic representation discussed here, the relation of these states with thermal states can be corroborated. As the result of this comparison, one can obtain certain thermodynamic properties such as the temperature of the system. This can also be extended for the bipartite harmonic oscillator, as seen in the next section.

## 6. Two-Mode Gaussian States in the Tomographic-Probability Representation

For a two-mode harmonic oscillator, the Gaussian-state tomogram is determined by the normal probability distribution of quadratures $X_{1}$ and $X_{2}$; it is expressed in terms of the state density operator $\hat{\rho }\left(1,2\right)$ as follows:

(68) $$w\left(X_{1},X_{2}|\mu_{1},\nu_{1},\mu_{2},\nu_{2}\right)=\mathrm{Tr}\left[\hat{\rho }\left(1,2\right)\;\delta \left(X_{1}-\mu_{1}{\hat{q}}_{1}-\nu_{1}{\hat{p}}_{1}\right)\;\delta \left(X_{2}-\mu_{2}{\hat{q}}_{2}-\nu_{2}{\hat{p}}_{2}\right)\right];$$

in the case of $\langle {\hat{q}}_{1}\rangle =\langle {\hat{q}}_{2}\rangle =\langle {\hat{p}}_{1}\rangle =\langle {\hat{p}}_{2}\rangle =0$, it reads:

(69) $$w\left(X_{1},X_{2}|\mu_{1},\nu_{1},\mu_{2},\nu_{2}\right)=\frac{1}{2\pi \sqrt{\det\;\sigma (\mu_{1},\mu_{2},\nu_{1},\nu_{2})}}\;\exp\left[-\frac{1}{2}\left(\tilde{X}\sigma^{-1}\left(\mu_{1},\nu_{1},\mu_{2},\nu_{2}\right)X\right)\right].$$

Here, $\;\tilde{X}=\left(X_{1},X_{2}\right)\;$ and $\;\sigma \left(\mu_{1},\nu_{1},\mu_{2},\nu_{2}\right)=\left(\begin{array}{cc} \langle X_{1}^{2}\rangle & \langle X_{1}X_{2}\rangle \\ \langle X_{1}X_{2}\rangle & \langle X_{2}^{2}\rangle \end{array}\right),$ with:

$$\begin{array}{c} \langle X_{1}^{2}\rangle =\mu_{1}^{2}\langle {\hat{q}}_{1}^{2}\rangle +\nu_{1}^{2}\langle {\hat{p}}_{1}^{2}\rangle +\mu_{1}\nu_{1}\left(\langle {\hat{q}}_{1}{\hat{p}}_{1}\rangle +\langle {\hat{p}}_{1}{\hat{q}}_{1}\rangle \right),\;\langle X_{2}^{2}\rangle =\mu_{2}^{2}\langle {\hat{q}}_{2}^{2}\rangle +\nu_{2}^{2}\langle {\hat{p}}_{2}^{2}\rangle +\mu_{2}\nu_{2}\left(\langle {\hat{q}}_{2}{\hat{p}}_{2}\rangle +\langle {\hat{p}}_{2}{\hat{q}}_{2}\rangle \right),\\ \;\;\;\;\langle X_{1}X_{2}\rangle =\mu_{1}\mu_{2}\langle {\hat{q}}_{1}{\hat{q}}_{2}\rangle +\nu_{1}\nu_{2}\langle {\hat{p}}_{1}{\hat{p}}_{2}\rangle +\mu_{1}\nu_{2}\langle {\hat{q}}_{1}{\hat{p}}_{1}\rangle +\mu_{2}\nu_{1}\langle {\hat{q}}_{2}{\hat{p}}_{1}\rangle . \end{array}$$

The inverse transform provides the density operator $\hat{\rho }\left(1,2\right)$ expressed in terms of the tomographic-probability distribution:

(70) $$\begin{array}{lll} \hat{\rho }\left(1,2\right) & = & \frac{1}{4\pi^{2}}\int w\left(X_{1},X_{2}|\mu_{1},\nu_{1},\mu_{2},\nu_{2}\right)\\ & & \times \exp\left[i\left(X_{1}+X_{2}-\mu_{1}{\hat{q}}_{1}-\nu_{1}{\hat{p}}_{1}-\mu_{2}{\hat{q}}_{2}-\nu_{2}{\hat{p}}_{2}\right)\right]\;dX_{1}\;dX_{2}\;d\mu_{1}\;d\mu_{2}\;d\nu_{1}\;d\nu_{2}. \end{array}$$

The subsystem tomogram given by the partial trace of the density operator $\hat{\rho }\left(1\right)={\mathrm{Tr}}_{2}\;\hat{\rho }\left(1,2\right)$ reads:

(71) $$w_{1}\left(X_{1}|\mu_{1},\nu_{1}\right)=\int w\left(X_{1},X_{2}|\mu_{1},\nu_{1},\mu_{2},\nu_{2}\right)\;dX_{2};$$

it is also given by the normal distribution discussed in the previous section.

If the tomogram of the two-mode oscillator state corresponds to the solution of the time evolution equation with a quadratic Hamiltonian, the unitary evolution of the system can induce the nonunitary evolution of tomogram (71). These evolutions can be used to obtain the shape of the invariant states, which we discussed above using the matrix M shown in Appendix B.

## 7. Summary and Conclusions

A differential formalism to obtain the time evolution of a multidimensional, multipartite Gaussian state was defined and studied. This new formalism used the time derivative of the parameters of the continuous variable density matrix of the system. The general procedure to obtain the time evolution can be summarized as follows: using the derivative of the covariance matrix for the Gaussian state and the expressions for the covariances in terms of the parameters of the density matrix, the differential equations for the parameters of the density function of the system were obtained. The resulting nonlinear differential equations could be used to obtain new physical information of the state instead of the use of the Schrödinger equation.

This differential formalism can also be used to describe exactly the nonunitary evolution of the subsystems of a composite Gaussian state. As an example, we considered a two-mode Gaussian state and demonstrated that the resulting derivatives of the covariance matrices for the subsystems contained unitary and nonunitary terms.

This study also allowed us to define invariant states, i.e., states that do not change their properties over time. To show this, we considered unimodal and bipartite Gaussian systems with density matrices in the position representation and the corresponding tomographic-probability representation. As explicit examples, we presented the invariant states for the one-dimensional quadratic Hamiltonian and the invariant states for the two-mode frequency converter and mentioned the applicability of these type of states in quantum information and computing. Furthermore, quasi-invariant states for the two-mode parametric amplifier were presented. We pointed out that the discussed examples of studying parametric systems could be used to apply the results associated with the behavior of physical systems like photons in cavities with time-dependent locations of boundaries to the dynamical Casimir effect (see [49]) and its analog in superconducting circuits [50,51]. One can discuss the nonunitary evolution of systems, which have no subsystems, using hidden correlations [52], which are present in noncomposite systems.

## Appendix A. Correspondence between the Gaussian Density Matrix Parameters and the Covariance Matrix

In this Appendix, the expressions of the covariance matrix and the mean values of the Gaussian system given in Section 4 are expressed in terms of the density matrix parameters. For the bipartite system, one can obtain the following formulas in terms of the density operator parameters (36) and the three covariances $\sigma_{q_{1},q_{1}}$, $\sigma_{q_{1},q_{2}}$, and $\sigma_{q_{2},q_{2}}$:

(A1) $$\begin{array}{lll} \sigma_{p_{1},p_{1}} & = & 2a_{11}-{\left(-2a_{11}+a_{13}\right)}^{2}\sigma_{q_{1},q_{1}}-{\left(a_{12}+a_{14}\right)}^{2}\sigma_{q_{2},q_{2}}+2\left(2a_{11}-a_{13}\right)\left(a_{12}+a_{14}\right)\sigma_{q_{1},q_{2}}\;,\\ \sigma_{p_{1},q_{1}} & = & i\{\left(2a_{11}-a_{13}\right)\sigma_{q_{1},q_{1}}-\left(a_{12}+a_{14}\right)\sigma_{q_{1},q_{2}}-1/2\}\;,\\ \sigma_{p_{1},p_{2}} & = & -a_{12}+\left(2a_{22}-a_{24}\right)\left(a_{12}+a_{14}\right)\sigma_{q_{2},q_{2}}+\left(2a_{11}-a_{13}\right)\left(a_{12}+a_{14}^{*}\right)\sigma_{q_{1},q_{1}}\\ & & -\left\{\left(-2a_{22}+a_{24}\right)\left(-2a_{11}+a_{13}\right)+\left(a_{12}+a_{14}^{*}\right)\left(a_{12}+a_{14}\right)\right\}\sigma_{q_{1},q_{2}}\;,\\ \sigma_{p_{1},q_{2}} & = & i\{\left(2a_{11}-a_{13}\right)\sigma_{q_{1},q_{2}}-\left(a_{12}+a_{14}\right)\sigma_{q_{2},q_{2}}\}\;,\\ \sigma_{q_{1},p_{2}} & = & i\{\left(2a_{22}-a_{24}\right)\sigma_{q_{1},q_{2}}-\left(a_{12}+a_{14}^{*}\right)\sigma_{q_{1},q_{1}}\}\;,\\ \sigma_{p_{2},p_{2}} & = & 2a_{22}-{\left(-2a_{22}+a_{24}\right)}^{2}\sigma_{q_{2},q_{2}}-{\left(a_{12}+a_{14}^{*}\right)}^{2}\sigma_{q_{1},q_{1}}-2\left(-2a_{22}+a_{24}\right)\left(a_{12}+a_{14}^{*}\right)\sigma_{q_{1},q_{2}}\;,\\ \sigma_{p_{2},q_{2}} & = & i\{\left(2a_{22}-a_{24}\right)\sigma_{q_{2},q_{2}}-\left(a_{12}+a_{14}^{*}\right)\sigma_{q_{1},q_{2}}-1/2\}\;, \end{array}$$

where the values of the rest of the covariances read:

(A2) $$\begin{array}{ccc} \sigma_{q_{1},q_{1}} & = & \frac{2(a_{22}+a_{22}^{*}-a_{24})}{4\left(a_{11}+a_{11}^{*}-a_{13}\right)\left(a_{22}+a_{22}^{*}-a_{24}\right)-{\left(a_{12}+a_{12}^{*}+a_{14}+a_{14}^{*}\right)}^{2}}\;,\\ \sigma_{q_{1},q_{2}} & = & \frac{a_{12}+a_{12}^{*}+a_{14}+a_{14}^{*}}{4\left(a_{11}+a_{11}^{*}-a_{13}\right)\left(a_{22}+a_{22}^{*}-a_{24}\right)-{\left(a_{12}+a_{12}^{*}+a_{14}+a_{14}^{*}\right)}^{2}}\;,\\ \sigma_{q_{2},q_{2}} & = & \frac{2(a_{11}+a_{11}^{*}-a_{13})}{4\left(a_{11}+a_{11}^{*}-a_{13}\right)\left(a_{22}+a_{22}^{*}-a_{24}\right)-{\left(a_{12}+a_{12}^{*}+a_{14}+a_{14}^{*}\right)}^{2}}\;, \end{array}$$

By the use of the time derivatives of the covariance matrix of Equation (34), it can be demonstrated that the density matrix parameters satisfy the following differential equations:

(A3) $$\begin{array}{lll} {\dot{a}}_{11} & = & i\omega_{22}-4\omega_{12}a_{11}+2\omega_{23}a_{12}+i\omega_{11}\left(-4a_{11}^{2}+a_{13}^{2}\right)+2i\omega_{13}\left(2a_{11}a_{12}+a_{13}a_{14}\right)-i\omega_{33}\left(a_{12}^{2}-a_{14}^{2}\right)\;,\\ {\dot{a}}_{22} & = & i\omega_{44}+2\omega_{14}a_{12}-i\omega_{11}\left(a_{12}^{2}-a_{14}^{*2}\right)-4\omega_{34}a_{22}+2i\omega_{13}\left(2a_{12}a_{22}+a_{14}^{*}a_{24}\right)+i\omega_{33}\left(-4a_{22}^{2}+a_{24}^{2}\right)\;,\\ {\dot{a}}_{12} & = & -2i\omega_{24}+4\omega_{14}a_{11}-2\omega_{12}a_{12}-2\omega_{34}a_{12}-2i\omega_{11}\left(2a_{11}a_{12}+a_{13}a_{14}^{*}\right)+4\omega_{23}a_{22}\\ & & +2i\omega_{13}\left(a_{12}^{2}-a_{14}a_{14}^{*}+4a_{11}a_{22}-a_{13}a_{24}\right)-2i\omega_{33}\left(2a_{12}a_{22}+a_{14}a_{24}\right)\;,\\ {\dot{a}}_{13} & = & -4\omega_{12}a_{13}-4i\omega_{11}\left(a_{11}-a_{11}^{*}\right)a_{13}-2\omega_{23}\left(a_{14}+a_{14}^{*}\right)+2i\omega_{13}(\left(a_{12}-a_{12}^{*}\right)a_{13}\\ & & +2a_{11}^{*}a_{14}-2a_{11}a_{14}^{*})+2i\omega_{33}\left(-a_{12}^{*}a_{14}+a_{12}a_{14}^{*}\right)\;,\\ {\dot{a}}_{14} & = & -2\omega_{14}a_{13}-2\omega_{12}a_{14}-2\omega_{34}a_{14}-2i\omega_{11}\left(a_{12}^{*}a_{13}+2a_{11}a_{14}\right)-2\omega_{23}a_{24}\\ & & +2i\omega_{13}\left(\left(a_{12}-a_{12}^{*}\right)a_{14}+2a_{13}a_{22}^{*}-2a_{11}a_{24}\right)+2i\omega_{33}\left(2a_{14}a_{22}^{*}+a_{12}a_{24}\right)\;,\\ {\dot{a}}_{24} & = & -2\omega_{14}\left(a_{14}+a_{14}^{*}\right)+2i\omega_{11}\left(a_{12}a_{14}-a_{12}^{*}a_{14}^{*}\right)-4\omega_{34}a_{24}+4i\omega_{33}\left(-a_{22}+a_{22}^{*}\right)a_{24}\\ & & +2i\omega_{13}\left(-2a_{14}a_{22}+2a_{14}^{*}a_{22}^{*}+\left(a_{12}-a_{12}^{*}\right)a_{24}\right), \end{array}$$

which can be used to determine the time evolution of the Gaussian system at any time.

## Appendix B. Matrix M for Bipartite System

As discussed in Section 4.2, the evolution of the covariance matrix of a bipartite system can be written as the linear system of equations:

Mv=0,

where the vector containing the different covariances reads:

$$\tilde{v}=\left(\sigma_{p_{1}p_{1}},\sigma_{p_{1}q_{1}},\sigma_{p_{1}p_{2}},\sigma_{p_{1}q_{2}},\sigma_{q_{1}q_{1}},\sigma_{q_{1}p_{2}},\sigma_{q_{1}q_{2}},\sigma_{p_{2}p_{2}},\sigma_{p_{2}q_{2}},\sigma_{q_{2}q_{2}}\right)\;,$$

and the matrix M is obtained by analyzing the derivatives of the covariance matrix; this results in an expression for M given by:

(A4) $$\begin{array}{c} M=\left(\begin{array}{cccccccccc} -4\omega_{12} & -4\omega_{22} & -4\omega_{23} & -4\omega_{24} & 0 & 0 & 0 & 0 & 0 & 0\\ 2\omega_{11} & 0 & 2\omega_{13} & 2\omega_{14} & -2\omega_{22} & -2\omega_{23} & -2\omega_{24} & 0 & 0 & 0\\ -2\omega_{14} & -2\omega_{24} & -2(\omega_{12}+\omega_{34}) & -2\omega_{44} & 0 & -2\omega_{22} & 0 & -2\omega_{23} & -2\omega_{24} & 0\\ 2\omega_{13} & 2\omega_{23} & 2\omega_{33} & 2(\omega_{34}-\omega_{12}) & 0 & 0 & -2\omega_{22} & 0 & -2\omega_{23} & -2\omega_{24}\\ 0 & 4\omega_{11} & 0 & 0 & 4\omega_{12} & 4\omega_{13} & 4\omega_{14} & 0 & 0 & 0\\ 0 & -2\omega_{14} & 2\omega_{11} & 0 & -2\omega_{24} & 2(\omega_{12}-\omega_{34}) & -2\omega_{44} & 2\omega_{13} & 2\omega_{14} & 0\\ 0 & 2\omega_{13} & 0 & 2\omega_{11} & 2\omega_{23} & 2\omega_{33} & 2(\omega_{12}+\omega_{34}) & 0 & 2\omega_{13} & 2\omega_{14}\\ 0 & 0 & -4\omega_{14} & 0 & 0 & -4\omega_{24} & 0 & -4\omega_{34} & -4\omega_{44} & 0\\ 0 & 0 & 2\omega_{13} & -2\omega_{14} & 0 & 2\omega_{23} & -2\omega_{24} & 2\omega_{33} & 0 & -2\omega_{44}\\ 0 & 0 & 0 & 4\omega_{13} & 0 & 0 & 4\omega_{23} & 0 & 4\omega_{33} & 4\omega_{34} \end{array}\right) \end{array}$$
